# The biological Maxwell's demons: exploring ideas about the information processing in biological systems

**DOI:** 10.1007/s12064-021-00354-6

**Published:** 2021-08-27

**Authors:** Eduardo Mizraji

**Affiliations:** Group of Cognitive Systems Modeling, Biophysics and Systems Biology Section, Facultad de Ciencias, Universidad de la República, Iguá 4225, 11400 Montevideo, Uruguay

**Keywords:** Biological Maxwell’s demons, Biological organization, Information and catalysis

## Abstract

This work is based on ideas supported by some of the biologists who discovered foundational facts of twentieth-century biology and who argued that Maxwell's demons are physically implemented by biological devices. In particular, JBS Haldane first, and later J. Monod, A, Lwoff and F. Jacob argued that enzymes and molecular receptors implemented Maxwell's demons that operate in systems far removed from thermodynamic equilibrium and that were responsible for creating the biological order. Later, these ideas were extended to other biological processes. In this article, we argue that these biological Maxwell's demons (BMD) are systems that have information processing capabilities that allow them to select their inputs and direct their outputs toward targets. In this context, we propose the idea that these BMD are information catalysts in which the processed information has broad thermodynamic consequences.

## Introduction

Understanding the link between thermodynamics and information has been one of the most challenging goals in natural science. Perhaps the search for this link began with Laplace and Maxwell's brilliant idea of approaching the difficulties of knowing the detailed dynamics of highly complex mechanical systems by statistical representations of collective behaviors. In the case of the study of statistical mechanics, this approach led to the microscopic interpretation of macroscopic variables such as temperature, pressure, viscosity or entropy. A corollary of this approach was the statistical interpretation of the increase in entropy in an isolated system.

In this context arose the famous thought experiment created by Maxwell around 1870. This experiment was described at the end of his "Theory of Heath," in the section called "Limitations of the Second Law." Let us quote this fundamental text (Maxwell 1871):

*... if we conceive of a being whose faculties are so sharpened that he can follow every molecule in its course, such a being, whose attributes are as essentially finite as our own, would be able to do what is impossible to us. For we have seen that molecules in a vessel full of air at uniform temperature are moving with velocities by no means uniform, though the mean velocity of any great number of them, arbitrarily selected, is almost exactly uniform. Now let us suppose that such a vessel is divided into two portions, A and B, by a division in which there is a small hole, and that a being, who can see the individual molecules, opens and closes this hole, so as to allow only the swifter molecules to pass from A to B, and only the slower molecules to pass from B to A. He will thus, without expenditure of work, raise the temperature of B and lower that of A, in contradiction to the second law of thermodynamics.*

This text gave birth to Maxwell's demon and from that distant 1871 to the present day this fictitious being stimulated a remarkable and extensive series of scientific contributions. A compilation of the most relevant contributions as well as a detailed bibliography has been edited by Leff and Rex (1990 and a revised edition in 2003).

The central interest of this imaginary experiment is that Maxwell found an intellectually challenging connection between thermodynamics and information, suggesting that information could be linked to entropy in a subtle way. The classic works of Szilard (1929) and Brillouin (1951) tended to show that the acquisition of information by the Maxwell's demon required increasing the entropy of this hypothetical being by an amount such that the global balance of the entropy change of the system [gas + demon] is positive and consistent with the second law of thermodynamics.

Starting in the 1960s, a new twist was produced on this topic that connected thermodynamics, information and logical operations of computing processes: The history and detailed references to this twist are in Leff and Rex (1990). In this line of research, the contributions of Landauer (1961) and Bennett (1982) emphasize the cost of erasing information and carry out a reinterpretation of the thermodynamics of a measurement system. In this framework, they review and partially try to replace previous theories regarding the thermodynamics of a hypothetical Maxwell's demon, although these new theories about the demon are not without criticism (see, for example, Porod et al 1984). We mention in passing, as a strange fact, the current coexistence of several physical theories that deal with the same topic, and in this sense, we refer the reader to the comprehensive reviews (see Lutz and Ciliberto (2015), and Parrondo et al. (2015)).

Maxwell's demon was initially an imaginary entity, but already in the late 1920s an idea emerged that Maxwell's demons could actually exist in living things and be promoters of the complex organization exhibited by individuals and biological collectives, in its various scales of complexity. These "biological Maxwell's demons" operate in open systems, in the midst of a wide availability of free energy and their role consists of channeling the energy transformations governed by information. The main promoter of this idea was JBS Haldane as a result of his research on the function of enzymes. Haldane raises the idea that enzymes are a physical realization of Maxwell's demons and how these devices operate using information to generate order (Haldane 1930). This initial idea of Haldane was taken up by other biologists, from whose work a good part of our understanding of the processes that regulate gene expression and fluxes in metabolic systems emerged, in particular André Lwoff, Jacques Monod and Francois Jacob.

The objective of the present work is to consider and to generalize Haldane's idea. In this article, even assuming the important technical and mathematical advances in the theory of Maxwell's demons, we deliberately want to place ourselves under the perspective of the aforementioned biologists, since we will assume that this perspective, stripped of the deep technicalities of contemporary theories, can show the existence of unsolved problems and stimulate new theoretical approaches.

We begin by showing how Haldane and other relevant researchers approached Maxwell's idea of demons materially implemented by biological objects. Then we will describe aspects of information processing in two categories of biological systems: enzymes and neural memories. We will also describe a thought experiment, which we will call "the parable of the prisoner," which highlights the complexity of the link between information and thermodynamics. Finally we show what the different biological Maxwell's demons have in common and propose the concept of information catalysts.

## Reviewing the original theoretical views on the biological Maxwell's demons

Unfortunately, the idea of the existence of Maxwell's biological demons, proposed by some of the researchers who participated in the creation of modern biology, was dispersed in various publications. This fact perhaps prevented the idea from being the subject of any in-depth research that would consolidate it in the scientific community.

The objective of this section is to quote some of these proposals about the real existence of Maxwell's demons in biology and its importance in the organization of living beings.

As far as we know, the first influential comment linking Maxwell's demons to properly biological objects was made by J.B.S. Haldane in his book "Enzymes" (1930, republished in 1965 by MIT Press). In this classic book, enzymes are analyzed as the fundamental catalyst molecules of cells, and in particular, their characteristic ability to select the reactions to be catalyzed is analyzed, as well as the basic kinetic equations. In addition, it is shown how these biological catalysts are subject to the laws of thermodynamics. In the opening chapter, Haldane notes:

* [...] if anything analogous to a Maxwell demon exists outside the textbooks it presumably has about the dimensions of an enzyme molecule and hence researches which show that the second law holds in the case of enzyme action possess a very general interest.*

For many decades now, it was known that the enzymes were macromolecules made up of chains of amino acids, with recognition sites for their ligands. Its basic action is to promote chemical reactions by reducing the activation energy, but without modifying the global thermodynamic properties of the reaction. An enzyme can only catalyze one thermodynamically possible reaction (Dixon and Webb 1979). Therefore, the cardinal property of enzymes is their ability to select which chemical reactions will actually occur within a cell, choosing from a myriad of potentially chemically and thermodynamically possible reactions. Thus, the set of enzymes in a cell operates as a filter that only retains a relatively few (actually thousands) chemical reactions out of the potentially possible total. Living beings are open systems, and the selection made by enzymes in natural cellular environments is immersed in a thermodynamic context where free energy abounds. This situation is the basis of the order that cells exhibit.

The idea of Haldane linking enzymes to Maxwell's demons was taken up by Norbert Wiener in his book "Cybernetics: Or Control and Communications in the Animal and the Machine" (Wiener 2019, on pages 83–84 of 2019 MIT Press edition). There Wiener writes,

*There is no reason to suppose that metastable demons do not in fact exist; indeed, it may well be that enzymes are metastable Maxwell demons, decreasing entropy, perhaps not by the separation between fast and slow particles but by some other equivalent process. We may well regard living organisms, such as Man himself, in this light. Certainly the enzyme and the living organism are alike metastable: the stable state of an enzyme is to be deconditioned, and the stable state of a living organism is to be dead. All catalysts are ultimately poisoned: they change rates of reaction but not true equilibrium. Nevertheless, catalysts and Man alike have sufficiently definite states of metastability to deserve the recognition of these states as relatively permanent conditions.*

(Let us mention, in passing, that Wiener had personal ties to Haldane, as detailed in the introduction to "Cybernetics").

It is important to note that the three researchers from the Pasteur Institute in Paris who discovered many of the control mechanisms of gene expression and cellular metabolism, André Lwoff, Jacques Monod and Francois Jacob, in various texts emphasized the identification between Maxwell's demons with enzymes and molecular receptors.

Lwoff in his book "Biological Order" (Lwoff 1962) focuses on the problem of the origin of biological order and its consistency with the laws of physics and uses Wiener's idea of enzymes as metastable Maxwell's demons:

*An organism is composed essentially of macromolecular compounds, among which are nucleic acids and proteins. Even the smallest organism contains a few thousand different species of macromolecules. The simplest organism is therefore a relatively complex machine. All known complex systems which contain macromolecules and are able to reproduce their kind belong to the living systems. Reproduction of a complex system containing macromolecules is therefore characteristic of life. And such a complex, independent unit of integrated structures and functions that reproduces true to type can only be an organism, a living organism. These statements might be considered too factual, and some would perhaps prefer a more original and sophisticated definition. The formulation which follows is an attempt at a summary of the views expressed by Norbert Wiener, in his fascinating book Cybernetics: "Living organisms are metastable Maxwell demons whose stable state is to be dead.*

Then Lwoff expands his book with an analysis of the details of biological organization and its ability to process information. Near the end of his book, in chapter VI, "Biological order and Entropy," he performs a deep synthesis of the facts behind the generation of the biological order and analyzes the notion of "negative entropy" presented by Schrödinger in his book "What is life?" (Schrödinger 1944).

In 1957, at the end of an influential research article describing the kinetic properties of permeases, a fundamental class of transporter proteins discovered in bacteria, the authors note (Cohen and Monod 1957):

*Enzymes are the element of choice, the Maxwell demons which channel metabolites and chemical potential into synthesis, growth and eventually cellular multiplication*.

The section of Monod's book "Chance and Necessity" that is devoted to the function of enzymes and their regulation is entitled "Maxwell's Demons" (Monod 1972a).

Let us highlight Monod's comment in his preface to The Collected Works by Leo Szilard, also published as an article in New Scientist in 1972. Referring to Szilard's simultaneous interest in Maxwell's demons and in the nature of living beings, he comments(Monod 1972b):

*Maxwell demons are, in fact, endowed with properties uniquely characteristic of living beings: choice, intention, and foresight. Yet, as Szilard showed, Maxwell demons, did they exist, would not, indeed could not, violate the principles of thermodynamics. Thus, in a very deep sense, the old dilemma of mind and matter at last receives its solution. The gap is bridged: the activity of the mind, expressed in an abstract thought, can organize matter without violating or superseding any physical principle.*

It is important that concerning Szilard interests, Monod then comments: *"[…] he was always a biologist at heart, and it is not accident that his last paper should be 'on memory and recall'."* In fact, during the investigation of enzyme induction in the lac operon by Monod and Jacob, a problem of difficult solution appeared, and it was Szilard who in a seminar proposed the idea of activation by double negation, which provided the key to solution.

Jacob discusses in detail the thermodynamic basis of biology and the role of enzymes as Maxwell's demons in his book "Logic of Life" (Jacob 1973). Furthermore, it extends this recognition capacity of enzymes to other categories of proteins with recognition abilities, such as gene repressors or neuronal receptors. Jacob writes:

* [...] proteins can, as it were, ' feel' the chemical species, 'sound ' the composition of the medium, 'perceive' specific stimuli of all kinds. They choose their associates because they 'know' only them. At all levels, proteins function like Maxwell's demons, fighting the mechanical tendency towards disorder. They hold the 'knowledge' by which the organization of the cell is maintained.*

These abilities of molecular recognition, ubiquitous in all living beings, expand and acquire unique and proper manifestations at various levels of complexity, including the function of sensory systems that inform individuals about properties of their environments and the cognitive capacities of neural networks.

In what follows, we will assume the reality of the physical existence of biological Maxwell's demons (BMD), but it should be clear that these BMD show a difference with Maxwell's demons of statistical mechanics. These were described for isolated systems in thermodynamic equilibrium (that is, under conditions of maximum entropy) and their hypothetical action caused asymmetries (thermal or material) that took the system out of equilibrium and, at first glance,  reduced entropy. In contrast, BMD at all scales (molecular to cognitive) operate in open systems, subject to a wide availability of free energy. However, let us assume here that the classic Maxwell's demons and BDM share a fundamental point in common: the ability to generate order.

It is well known that in an open system, structures can be formed that involve a local reduction in entropy at the expense of a global increase in the entropy of the "environment–system" coupling. A pioneering example that illustrated this was the Turing's ring, a reaction–diffusion system proposed as a stylized model of biological morphogenesis based on "morphogen" gradients (Turing 1953). The Turing model implicitly imposed a free energy input in the ability of membranes to carry out morphogen transports against concentration gradients. A famous experimental case that illustrates the complexities of chemical reaction–diffusion systems is the Belousov–Zhabotinsky reaction (Winfree 1984). These processes generate ordered patterns of medium complexity. On the other hand, the set of BMD determines, in an open system with wide availability of free energy, that the biological system remains in a state of very high complexity and order, as described in the book by Lwoff (1962). Other important analyzes on the nature of the complexity of living things were carried out by Hopfield (1994), Kauffman (2020) and Pattee (1969, 1973). A formal treatment of the constraints present in highly complex systems that includes individuals, groups or organizations was carried out by Madden and Ashby (1972); Montévil's research (2021) is framed in the same area of very complex systems.

These BMD create order by their ability to recognize and select patterns, and without ever violating the laws of thermodynamics, they have much broader thermodynamic consequences than the energy cost of their construction. Thus, for example, the energy cost of the synthesis of an enzyme is not immediately related to the thermodynamic consequences of the reactions that that enzyme catalyzes. Similarly, the cost of consolidating a neural memory is not related to the energetic consequences of using the data in that memory. We will illustrate this in the imaginary experiment (the parable of the prisoner) described in Sect. 4.

## The "molecular neural" shift and the ubiquity of BMD

It is interesting to note the fact that some of the great names in modern science have shifted the focus of their scientific interest from the recognition capabilities of molecular systems to the processing of information by neural networks. These shifts have historical as well as epistemological interest.

Norbert Wiener in his book "Cybernetics," after proposing that enzymes are metastable Maxwell's demons and after describing his own mathematical theory of information, has a chapter entitled "Gestalts and Universals." There, he adopts Locke's philosophical stance on the association of ideas to elaborate a neural mechanism and develops a theory of the creation and association of concepts (the "universals"), based on sensory inputs; in his analysis, he includes the possible creation by engineering methods of sensory prostheses (Wiener [1948] 2019).

Szilard's article on neural systems, mentioned by Monod in the previous section citation, is titled "On Memory and Recall" (Szilard 1964). In the 1950s, Szilard had worked on problems associated with bacterial metabolism (Novick and Szilard 1950).

Monod, in Chapter 8 of "Chance and Necessity" states that central nervous system research is one of the frontiers of knowledge. There, Monod writes: "*An understanding of the central nervous system's functioning must begin with that of the synapse, its primary logical element. Investigation is easier here than at any other level, and refined techniques have yielded a considerable mass of findings. However, we are still a long way from an interpretation of synaptic transmission in terms of molecular interaction. Yet that is a most essential question, for therein probably lies the ultimate secret of memory.*"

Jean-Pierre Changeux follows this path indicated by Monod, and having established the true molecular nature of allosteric enzymes (Changeux 1962), he concentrates on the study of the complex properties of the acetylcholine receptor. Finally, Changeux focuses on the cognitive properties of the human brain and neural modeling (see, for example, Gisiger et al. 2000); a summary of this trajectory is described in his book "L'Homme Neuronal" (Changeux 1983).

We now describe an interesting parallel between two researchers on the "molecular neural" shift. In 1974, John Hopfield published a highly influential article on kinetic proofreading, a procedure by which enzymes that replicate genetic material correct errors intrinsic to the physicochemical process. This kinetic proofreading is a process that required free energy consumption (Hopfield 1974). Independently, Jacques Ninio, in France, publishes a kinetic model of amplification of the fidelity of the enzymatic action when replicating the genetic material (Ninio 1975). This procedure presented by Nino was, except for differences in formalism, equal to that presented by Hopfield, so that from that date on both articles are usually cited together. In 1982, Hopfield publishes a paper that creates a new paradigm in the research of neural memory models and then extends the study of neural systems in various directions (Hopfield 1982, 2010). As for Ninio, after his research in molecular biology, he concentrates the investigation on visual perception (among his many contributions, see, for example, Ninio and Mizraji 1985) and then focuses on the neural models of memories (Ninio 1996)

We are going to finish this description of the "molecular neural" shift by showing two cases of special importance. Gilles de Gennes was a physicist specializing in various areas of materials physics. When he won the Nobel Prize in 1991, the Academy stated the reason for the award "for discovering that methods developed for studying order phenomena in simple systems can be generalized to more complex forms of matter, in particular to liquid crystals and polymers." Several years before, Monod presented de Gennes with a problem of vital importance: Is it possible to determine the minimum length necessary for a polypeptide chain to generate, when folding, a hole with stereochemical recognition capacity, as in the active sites of enzymes? De Gennes's important solution was first published at a symposium, and much later, he included it in a book (de Gennes 1990, chapter 2 ["Minimum number of aminoacids required to build up a sprcific receptor with a polypeptyde chain"]). De Gennes's intellectual concerns were extremely broad, and in this sense, we note that one of the de Gennes's last publications was a neural model to explain the organization of a neural memory system (de Gennes 2004).

The last case we will deal with is that of Gerald Edelman. Following his research on the structure of antibody molecules (for which he was awarded the Nobel Prize in 1972), Edelman focused on the study of molecular recognition systems that partly supported embryonic development, and his group studied fundamental molecular family, related to antibody molecules, called cell adhesion molecules (CAMs) (Edelman 1983). Then Edelman created a group dedicated to the study of neural systems using mathematical and computational analysis (Edelman and Reeke 1982). Perhaps his most comprehensive work in this area is his book "The Remembering Present: A Biological Theory of Consciousness" (Edelman 1989) where he presents an elaborate anatomical and neurodynamic theory of conscious activity.

Based on this scientific interest in connecting molecular pattern recognition with neural cognition, in the next two subsections we will illustrate ideas about BMD using both levels of complexity.

### Enzymes and receptors as molecular BMD

At this point, we can consider the mode of action of biological catalysts and molecular receptors.

In living cells, enzymatic reactions usually occur under conditions in which there is a gradient of chemical potential between substrates and products, which defines the direction of the reaction. However, as noted before, enzymes do not modify the thermodynamic equilibrium of the reaction they catalyze. Its central function is the reduction of the activation energy of the reactions. This situation is what highlighted, in the early days of the development of enzymology, the importance of the Haldane relations derived by this researcher when investigating the kinetics of reversible reactions (Haldane 1930). Given the historical importance of this, we include in "Appendix 1. Haldane relation" the deduction of the Haldane relation for the simplest situation, in which a single substrate is transformed into a single product. Wyman (1975) published an important article that analyzes enzyme systems that operate through cycles that occur very far from thermodynamic equilibrium and show the need for the kinetic constants of these cycles to satisfy the conditions of microscopic reversibility (Onsager 1931 a, b). As a consequence, a kinetic model that "disobeys" this postulate violates thermodynamics and generates chemical work without an adequate source of energy, so it must be rejected.

**Fig. 1:**
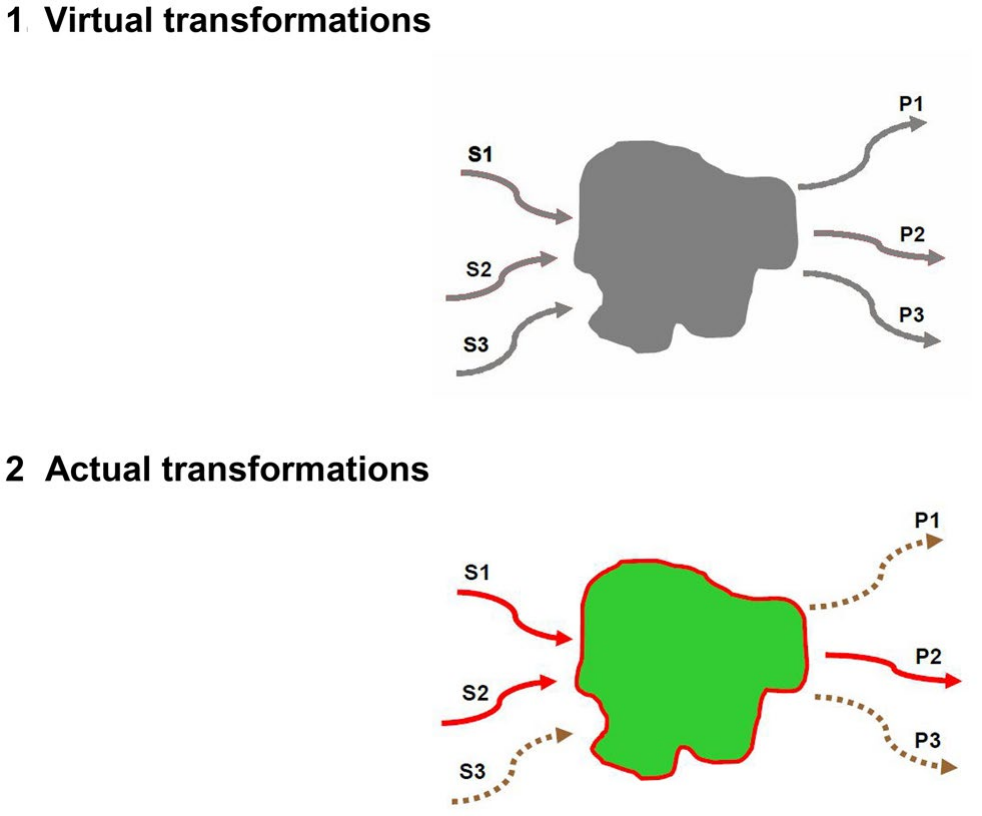
Panel (1) shows the potentially combinable reagent set in gray. Panel (2) shows current reactions with arrows in solid lines in contrast to non-catalytic ones (dashed gray line) (Color figure online)

We are now going to illustrate in the following model enzyme system a cardinal property of molecular BMD, namely their ability, due to the specificity of their receptors, to (1) select their substrates and reduce the initial variety of a broad set of potential substrates and (2) to channel reactions into a well-defined set of products. Suppose that three substrates *S*_1,_ *S*_2_ and *S*_3_ exist in an intracellular medium with the thermodynamic possibility of isomerizing or combining to produce the products *P*_1,_*P*_2_ and *P*_3_. In this situation, let us imagine that there is an enzyme E capable of catalyzing the combination of *S*_1_ and *S*_3_to synthesize *P*_2_. This reaction is one of several potential (and not necessarily possible) reactions; For example, perhaps the combination of *S*_1_and *S*_3_ to give *P*_1_ is also thermodynamically legal, but there is no catalyst that allows reducing the activation energy barrier. But the presence of the enzyme E is what determines that only the combination of *S*_1_ and *S*_2_ synthesizing *P*_1_ can occur under intracellular conditions. Figure 1 illustrates this situation.

We can see this catalysis as a selection and channeling process, where the selection depends on the association constants *K*_A1_ and *K*_A2_ respect to the main substrates and the channeling toward a goal is represented by the catalytic constant *k*cat associated with the rate of synthesis of* P*_2_.

In a realistic situation, the cell exhibits hundreds of substrates and hundreds of thousands of reactions. Existing enzymes are the result of genetic coding and the number of genes in a complex organism such as humans is less than 30,000 (Venter et al 2001). A few years ago, the panorama has broadened after it was shown that, via splicing of its mRNA, a gene can support the synthesis of several different polypeptide chains, capable of participating in different enzymatic activities (Yang et al. 2016). However, the variety of enzymes is much less than the potential variety of thermodynamically possible reactions. This implies that enzymes exert a dramatic reduction in the space of possible reactions and that they are specialized in that subset of reactions selected during evolution and consistent with the adequate adaptations to achieve acceptable conditions of survival. It is this ability to promote drastic selection through preexisting information at their active sites and the power to catalyze toward prefigured targets, which makes enzymes the most basic BMD.

### Associative memories as cognitive BMD

Let us now go to the other extreme of the levels of complexity of the individual and consider some extremely simple models of the way in which the nervous system of an animal (to fix ideas, think of the brain of a human being) stores and processes information.

Neural memories are pattern associators. In the human nervous system (and in many other kinds of animals), these patterns are representable as vectors of real, high-dimensional components (Mizraji et al. 2009). Let us illustrate this: An image captured by a retina is transported to the human brain by electrochemical signals (the actual components of the vector) carried by around 10^6^ axons that penetrate the brain. This is what is naturally conceived as a neural vector: in this case an array of around 10^6^ real components. This neural vector after being processed in a succession of nerve centers becomes another vector that triggers a cognitive decision about the known or unknown nature of the perceived object. If the perceived object was the face of a friend, then some memory could associate its corresponding vector with another neural vector that represents the name of that friend.

The use of a neural coding by means of real vectors of large dimension and the existence of memories that associate vector patterns is generalizable to many of the events of the cognitive life of complex animals, including the human being.

Let us point out that the selection capacity of these memories is enormous. A memory Mem with K pairs of associated vector patterns (f, g) is a restriction arising from an enormous possible combinatorial variety of vectors of the same dimension as f and g. Usually

$${\rm cardinal(Mem)} \ll {\rm cardinal}\left[ {\left\{ {f \in {\mathbb{R}}^{m} } \right\} \times \left\{ {g \in {\mathbb{R}}^{n} } \right\}} \right]$$

where × is the Cartesian product. The cognitive functions of the brain are the result of the interaction of modules that support these neural BMD. The following example illustrates this point.

Let us suppose a memory C that can associate a sequence of inputs (e.g., the Morse code symbols) represented by vectors *s*_k_, to their literal or numeral meaning, represented by vectors *a*_k_(k runs from 1 to K). So a decoding of a symbol *s*_i_ is represented by

$$Cs_{i} = a_{i.}$$

At the same time, suppose that this interpretation *a*_i_ is the input of a neural memory M that associates it with a motor act *m*_i_ (e.g., writing the decoded symbols or using the numbers to open a combination box). So

$$Ma_{i} = m_{i} .$$

**Fig. 2:**
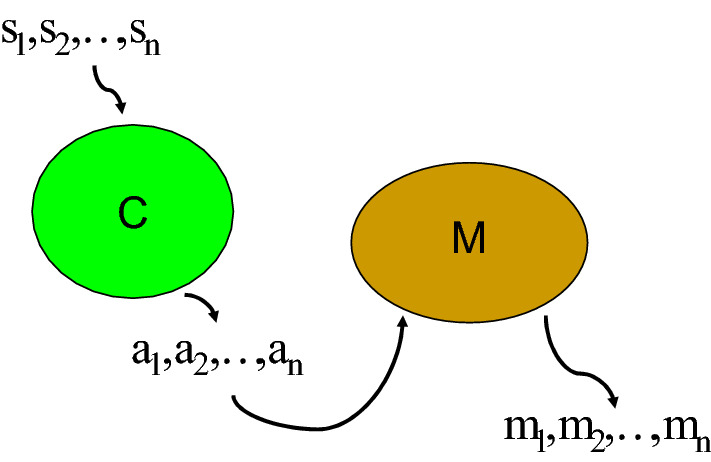
It shows how, through previously trained associations, a succession of abstract symbols (for instance, the dots and dashes of Morse code) end up generating a succession of motor acts

This pair of memories (certainly simplifying the neural and motor reality to the maximum) can give an idea of how a succession of abstract symbols becomes a succession of motor acts. This is illustrated in Fig. 2.

These simplified neural models illustrate the basic elements of the design of any neural configuration that performs complex cognitive activities. All these systems have a design prepared for the selection of specific patterns and the association of each pattern toward a particular target.

## Energy and information: the parable of the prisoner

In the next section, we will refer to several important insights into the meaning of "information" developed by different researchers for different situations. Here we want to emphasize that there is certainly an important interplay between the meaning of information and the laws that govern the physical (including biological) world. This interaction is in fact the basic reason that allows the operation of the BMD. But this interaction probably still has regions that need clarification. Let us describe in parable form a fictional situation that illustrates this interaction.

Let us imagine that a person is locked up in a prison whose door is closed by a clockwork mechanism that will open it automatically three months after the lockdown begins. Here is a rudimentary bathroom and the cell is continuously ventilated; there is never a lack of air. But the prisoner has no access to water or food. Perversely, his captors have stored the water and canned food necessary for him to survive during his captivity, in a safe box located inside the cell and locked with a combination lock. It also has a window with an unbreakable glass that allows the prisoner to see the outside. In addition, through the window, signals of light arrive continuously. These signals are emitted with a constant power (i.e., with the same energy expenditure per unit of time). But the light signals are a mixture of random sequences with sequences coded in Morse that gives the prisoner the key to open the security lock. As we will see next, if the way to decode the signals in Morse is not in the prisoner's memory, his death is unavoidable. Hence, the two possible scenarios are the following:

- a) In the first scenario, if the prisoner cannot extract any significant data, time passes, he never manages to open that box, and the story has an ugly and tragic end, imposed by the cruel indifference of the laws of thermodynamics.
- b) In the second scenario, the prisoner recognizes signals in Morse, decodes its meaning, and he goes to the box and opens it; there is the water and canned food that allow him to survive three months, after which the door opens automatically and he returns alive to the world.

It is his cognitive ability to filter and recognize patterns of external information, which gives the prisoner access to water and food. Thus, the locked man can transfer the energy from these foods to his body, hydrate himself and survive. But here an enigmatic fact arises: decoded information can trigger very different thermodynamic fates (see "Appendix 2. Survival modes").

Let us also note that the “meaning” of the signal arises from the interaction between the selection of well-configured messages and the receiver who knows the code. We will analyze in more detail the situation described by this parable in the next section.

## Catalysis by information

The idea that life acts globally as a catalyst has been proposed early by Leon Brillouin in his famous article published in American Scientist (Brillouin 1949). Here we try to develop this idea assuming that the executors of this catalytic capacity of living beings are the BDM and that the physical basis of their catalytic capacity resides in the filtering and channeling characteristics described in the previous sections.

The parable of the prisoner illustrates how information, depending on the organization of the prisoner's memory, can generate drastically different physical processes. This suggests a general principle that we will illustrate for the two cases previously studied: information processing by enzymes and molecular receptors, on the one hand, and by associative memories on the other.

Let us start by noting that the notion of information is dependent on the context in which it is used. In addition to the classic definition of "amount of information" proposed by Shannon (1948), the Wikipedia article "Information" (June 6, 2021) lists a variety of conceptions of information in different domains. An interesting example is provided by Ashby's (1963) analysis of the notion of "information" in a biological and bioengineering context. We mention in this framework the notions of representational information and structural complexity developed by Vigo (2011, 2013).

Many investigations have been developed trying to capture the various information processing procedures carried out by natural or artificial systems. This led to complement the combinatorial (or syntactic) theories of information with theories that focus on the meanings of the data, i.e., on the semantics of the information. With this objective, Deacon has published a broad unifying vision of territories where the notion of information requires the analysis of patterns and actions (Deacon 2007). Likewise, this author has also investigated the links between information and language (Deacon 2008). A detailed mathematical theory on semantic information has been published by Kolchinsky and Wolpert (2018). Another objective of the researchers, linked to the previous point, has been the search for a representation of the value of the information. The pioneering works on this point was carried out by Woodward (1953) and Kharkevich (1960, also written Kharkevits or Jarkievich). These authors propose to measure the value of information by its ability to contribute to reaching targets. This value of the information is measurable (for example in bits), and it will be positive if the data increases the probability of accessing a target, with respect to the basal probability represented by a random search. On the contrary, this measure can give a negative value if the data "misinforms" and reduces the probability of access to the target. A use of the Kharkevich measure in a biological situation is found in Mizraji (1985). Recently, Montévil (2021) has published an investigation on highly complex systems generated by human societies and their multiple activities, which leads him to expand the thermodynamic framework to describe and analyze the dynamics of these systems. In this expansion, Montévil uses the notion of "anti-entropy" (Bailly and Longo 2009; Longo and Montévil, 2014, Ch.9) to describe the coupling between biological organizations and their environment, where the local decrease in entropy is subject to strong constrictions. In his 2021 article, Montévil analyzes the dynamics that can lead to the disruption of the structure of organizations, rarifying anti-entropy (a situation called "Anthropocene Crisis").

*In the present context, we will define "information" as an emergent property that arises from an operator that acts as a pattern selector for a system.* In the enzymes and molecular receptors, we had represented their capacities for recognition and action. In associative memories, recognition and action are also the main characteristics of its performances. In both cases, we have biological objects whose existence demands a cost of construction and eventually of operation (e.g., enzymes that carry out active transports, or in the case of memories, the cost of transporting bioelectric signals). All the properties of these devices are the consequence of being immersed in an open thermodynamic system and not being limited by energy consumption.

On each scale the BMD operate for many cycles of transformations until finally, they too, subject to the second law of thermodynamics, end up deteriorating (this is the "metastability" mentioned by Wiener). In all cases, the information from the BMD catalyzes transformations that have very diverse consequences that can be energetic, behavioral, cultural, technological or environmental.

Let us symbolize an information catalyst by iCat. Any iCat is defined by a pair of filters $\Im$, one selecting the input and the other channeling the output. In symbols, we express the idea with this equation:

$$iCat = \left[ {\,\Im_{input} \circ \,\,\Im_{output} } \right].$$

In this framework, each transition $[(Pattern) \to (Target)]$ is performed by the functional composition of the associated operators $\Im$. The catalytic nature of the various iCats arises from the fact that once a biological Maxwell's demon executes an action dependent on its $\Im_{input}$ and $\Im_{output}$ operators, the iCat is ready for a new cycle of action, similarly to the classic catalysts of chemistry.

Is it always possible to set the symbolic equivalence iCat = BMD? The validity of this equivalence depends on the criteria we use to define a biological system. Let us note that the notion of information catalysis should not necessarily be restricted to natural objects. For example, take the case of a Fredkin gate (Fredkin and Toffoli 1982). This is a reversible artificial logic device capable of computing various monadic and dyadic logic operations. But its ability to compute depends on the existence of selector filters of the inputs and selector filters of the output channels, similar to the $\Im_{input}$ and $\Im_{output}$ shown before. (see details in Mizraji 2008). The Fredkin gate is not, in appearance, a biological object. However, it has been devised by a human mind, and in that sense, the biology of its designer's brain is implicit in design. In this regard, it is interesting to consider the visions of Louis Rapkine and Jacques Monod on the biological nature of art and, consequently, esthetics (Rapkine 1970; Monod 1970).

We could say that the information processed by an iCat generates a transition between a selection area of patterns in the input and a selection area of actions in the output, where each selection area is represented by its corresponding $\Im$. The following is a diagram of this information catalysis:

$$filtered\,\,input\,\,\,\mathop{\longrightarrow}\limits^{i\,Cat}\,\,\,channeled\,\,output.$$

To illustrate this idea in a complex situation, let us return to the parable of the prisoner. The prisoner can decode the message if the light is formatted according to Morse code. *But in order to decode the formatted signal he must have the code keys in his memory. This is a crucial point in the recognition of patterns and it is repeated at all levels of complexity.* Let us also point out that a pattern that transfers meanings (such as a region of a molecule or the Morse code) is not a random signal but a configuration that arises through a long evolutionary process. For both an enzyme and a brain, the pattern can be recognized only if its structure preexists represented in the receptor. This representation depends on the nature of the situation. Jorge Luis Borges (1985) expressed this idea in an elegantly compact way in the dedication of "Los Conjurados," his latest book of poems: *"We can only give what is already in the other"* ("Sólo podemos dar lo que ya es de otro"). For a similar concept, see the last paragraph of Monod's note (1970).

In the case of the parable of the prisoner, the cognitive situation is similar to that represented by Fig. 2, in that a succession of inputs recognized by the neural receptor system triggers a succession of responses (the actions that lead to the opening of the safe box). This complex process can be assigned to the neural information catalyst

$$iCat_{neural} = \left[ {\,\Im_{input}^{C} \circ \,\,\Im_{output}^{M} } \right]$$

where the $\Im_{input}$ corresponds to the neural module C that contains information on how to decode Morse code and it is functionally composed with the $\Im_{output}$ that corresponds to the neural module M that executes the succession of motor acts that lead to open the box. However, an individual without information about the code in his memory C does not have the $\Im_{input}$ operator that triggers the informational catalysis. That faced with this flow of optical information, the individual's nervous system can act as a BMD, basically depends on his memory building an $\Im_{input}$ operator and also on the ability to execute the correct movements to reach the goal, corresponding to the existence of the operator $\Im_{output}$. This hypothetical situation illustrates the close link between pattern recognition and target access. A mathematical formalism is included in "Appendix 3. Towards a formal representation of Information Catalysis", Appendix that describes how an iCat works.

In short, the parable of the prisoner illustrates the different thermodynamic consequences caused by knowing or not knowing the code. Not knowing the code leads to death and the increase in entropy that it entails and which will depend on the physical characteristics of the individual. On the other hand, knowing the code postpones death and generates variable survival lapses that lead to different energy consumptions and the entropy production associated with the normal life processes (see "Appendix 2. Survival modes"). This raises an enigmatic aspect of the link between information and energy that may merit further investigation.

## Conclusions and perspectives

The ability of BMD to construct order at the various scales in which they act (as Brillouin, Lwoff and Jacob pointed out at the time) is the consequence of a large set of changes generated and selected during millions of years of biological evolution. The consequent functional refinements exhibited by genes, enzymes, cells and organs appear to be oriented toward goals that contribute to the stability of individuals in their natural environments. The possession of apparent targets shown by the biological functions in all their scales has been called "teleonomy"(Monod 1972a). Teleonomy is an attribute of objects that appear to have a target-oriented design, but created without a designer. BDM are the result of refinements accumulated and retained during geological time by the mechanisms of evolution and natural selection. Some BMD can be adequately described by physical or mathematical representations. The examples in the previous sections showing partial aspects of enzyme kinetics and associative memory theory illustrate forms of representation of BMD.

Biological Maxwell's demons carry out specific information processing in highly complex systems, and they are placed very far from thermodynamic equilibrium and deteriorate after several cycles of catalytic action. These deteriorated information catalysts can be replaced in a number of ways. Impaired enzymes in a cell are replaced by the synthesis of new enzyme molecules, which sometimes remain in a dynamic steady state as long as the  molecule is viable. In the case of associative memories, there may be various forms of substitution, either by new synthesis of synaptic receptors or by the replacement of the function of a memory impaired by another neural module.

When individuals die, some important cognitive performances that disappear with individuals may remain indirectly in the memory of other people, as well as in a vast variety of instruments, in artistic and architectural productions, and also in libraries, or other databases. All of this configures the cultural legacy that our civilization preserves today based on the achievements made by people who died centuries ago.

Physical theory of Maxwell’s demons has undergone remarkable advances, some of which are mentioned in the Introduction. But the link between information and thermodynamics displayed in biological systems may reveal significant problems that challenge the development of adequate physical theories.

## Appendix 1. Haldane relation

Here we will see an elementary derivation of the Haldane relation. Enzymes help chemical reactions proceed under conditions of microscopic reversibility (Onsager 1931). A kinetic model that violates this thermodynamic principle would be able to generate chemical work without an adequate source of energy.

Haldane (1930)  seems to be the first scientist to publish the kinetic equation for a reversible enzymatic chemical reaction,

$$S\,\,\,\overset {} \rightleftharpoons \,\,\,P.$$

In cellular environments, this reaction cannot usually proceed without the presence of an enzyme (same letters are used to designate chemical species concentrations). The enzyme E creates an intermediate complex X.

$$E + S\,\,\underset{{k_{ - 1} }}{\overset{{k_{1} }}{\rightleftharpoons}}\,\,\,X\,\,\,\underset{{k_{ - 2} }}{\overset{{k_{2} }}{\rightleftharpoons}}\,\,\,E + P,$$

where $k_{i\,} {\text{and}}\,k_{ - i} ,i = 1,2$ are the forward and backward kinetic constants. In an experimental closed system, this reaction ends at equilibrium. In open cellular environments, the substrate is injected– usually as a product of other reactions–and the product P joins in other parallel reaction paths, thus making the whole process practically irreversible.

This equation from Haldane (1930) was derived assuming the steady state of complex X. The rate v of product changes ($v = {\text{d}}P/{\text{d}}t$) is written as

$$v = \frac{{k_{1\,} k_{2\,} S\, - \,k_{ - 1\,} k_{ - 2} \,P}}{{k_{ - 1} + k_{2} + k_{1} \,S + k_{ - 2} \,P}}\,\,E_{{{\text{T}}\,}} = \frac{{V_{1} \left( {S/K_{1} } \right) - V_{2} \left( {P/K_{2} } \right)}}{{1 + \left( {S/K_{1} } \right) + \left( {P/K_{2} } \right)}}.$$

with $$V_{1} = k_{2} E_{T} S$$, $$V_{2} = k_{ - 1} E_{T} P$$, $$K_{1} = \left( {k_{ - 1} + k_{2} } \right)/k_{1}$$ and $$K_{2} = \left( {k_{ - 1} + k_{2} } \right)/k_{ - 2}$$.

The total concentration of enzyme is $E_{{{\text{T}}\,}}$ and *S*, and *P* are the concentrations of substrate and product. At equilibrium, v = 0, and the kinetic constants obey microscopic reversibility,

$K_{{{\text{eq}}}} = \left( {\tfrac{P}{S}} \right)_{{{\text{eq}}}} = \frac{{k_{1\,\,\,\,} k_{2} }}{{k_{ - 1\,} k_{ - 2} }}$, and consequently, we have $K_{{{\text{eq}}}} = \frac{{V_{1} \,K_{2} }}{{V_{2} \,K_{1} }}$.

This last beautiful expression is known as the "Haldane relation," and it is a strong argument to show the thermodynamic consistency of enzymatic catalysis.

Note that *K*_1_ and *K*_2_ can be expressed in terms of the association constants *K*_S_ and *K*_P,_ on which the recognition of ligands S and P by the receptor sites depends. The respective equations are:

$$K_{1} = \frac{{k_{ - 1} + k_{2} }}{{k_{1} }} = \frac{1}{{K_{{\text{S}}} }} + \frac{{k_{2} }}{{k_{1} }},$$

$$K_{2} = \frac{{k_{ - 1} + k_{2} }}{{k_{ - 2} }} = \frac{1}{{K_{P} }} + \frac{{k_{ - 1} }}{{k_{ - 2} }}.$$

Very far from equilibrium, $S \gg P$, rate v becomes the classical Michaelis–Menten equation,

$$v \approx V_{1\,} \,S/\left( {K_{1} + S} \right).$$

## Appendix 2. Survival modes

Here we want to discuss an interesting point related to the physical effects of the presence of the iCat. Let us imagine that the emission energy of the light signal is in both situations $\varepsilon_{{\text{E}}}$. Suppose that formatting the emission of that light to transfer the information in Morse requires an additional energy $\varepsilon_{{\text{F}}}$. Finally suppose that the prisoner's decoding the Morse code has consumed an energy $\varepsilon_{{\text{L}}}$. Then, the conditions associated with the code that allow the prisoner to open the combination box, access the food and save his life have a total energy cost $\varepsilon_{{\text{T}}}$ given by

$$\varepsilon_{{\text{T}}} = \varepsilon_{{\text{E}}} + \varepsilon_{{\text{F}}} + \varepsilon_{{\text{L}}} .$$

Let us now expand the experiment to Q isolated prisoners, trapped in their cells and released at age $m_{i}$, i = 1, …, Q. Suppose everyone knows Morse code, gets saved and continues their lives. If these i individuals live a total of $n_{i}$ years, each one of them will carry out a total consumption of free energy given by

$$F_{i} \left( {m,n} \right) = \int\limits_{{m_{i} }}^{{n_{i} }} {W_{i} (a)\,{\text{d}}a} ,$$

where $W_{i} (a)$ is the instantaneous power expressed in units of age. It is expected that in general for each pair of individuals j, k, it will be $F_{j} (m,n) \ne F_{k} (m,n)$. This implies that the information associated with a relatively small amount of energy, $\varepsilon_{T}$, generates different amounts of dissipation of free energy $F\left( {m,n} \right)$. It is also expected to be $F(m,n) \gg \varepsilon_{{\text{T}}}$ and that both amounts are not correlated.

## Appendix 3. Toward a formal representation of Information Catalysis

The information catalyst iCat is built from two systems of pattern recognition, the operators $\Im_{{{\text{input}}}}$ and $\Im_{{{\text{output}}}}$. As we previously pointed out, the semantic information processed by an iCat is located (or, we could also consider, "defined") in the structure of its filters $\Im_{{{\text{input}}}}$ and $\Im_{{{\text{output}}}}$.

Let us start by describing the way these iCats work. In a transition between an initial state $Y_{ \downarrow }^{{({\text{in}})}}$ and a final state $Z_{ \uparrow }^{{({\text{fin}})}}$(represented by $Y_{ \downarrow }^{{({\text{in}})}} \mathop{\longrightarrow}\limits^{{}}Z_{ \uparrow }^{{({\text{fin}})}}$) with very low probability $p_{0}^{{({\text{in,fin}})}} \approx 0$ of occurrence, a catalyst iCat guides the same transformation $Y_{ \downarrow }^{{\text{(in)}}} \mathop{\longrightarrow}\limits^{{{\text{iCat}}}}Z_{ \uparrow }^{{({\text{fin}})}}$, with a transition probability $p_{{{\text{iCat}}}}^{{({\text{in,fin}})}} \gg p_{0}^{{({\text{in,fin}})}}$. The subscripts $\downarrow$ and $\uparrow$ mean non-filtered and filtered, respectively. After the transition is completed, the catalyst recovers its ability to initiate a new cycle. It is important to remark that iCat, instead of enhancing rates as it happens in chemical catalysis, drastically increase the probabilities of occurrences of the involved events***.***

The states Y and Z have a very variable inner structure depending on the patterns involved. We can assume the following symbolic representation of the action of the filters of iCat:

$$Y_{ \downarrow }^{{{\text{(in}})}} \mathop{\longrightarrow}\limits^{{\Im_{{{\text{input}}}} }}Y_{ \uparrow }^{{({\text{in}})}} \,{\text{and}}\,Z_{ \downarrow }^{{({\text{fin}})}} \mathop{\longrightarrow}\limits^{{\Im_{{{\text{output}}}} }}Z_{ \uparrow }^{{({\text{fin}})}}$$

Consequently, a large set of potential states $Y_{ \downarrow }^{{({\text{in}})}}$ is filtered to a selected subset of actual states $Y_{ \uparrow }^{{{\text{(in}})}}$ (e.g., the selection of a particular ordered set of substrates of an enzyme form the large set of potential substrates), and a large set of potential states $Z_{ \downarrow }^{{({\text{fin}})}}$ is filtered to a selected subset $Z_{ \uparrow }^{{({\text{fin}})}}$ that gives the final outcome (e.g., for the case of an enzyme, and the filter reduced the set of potential products to a set of actual products).

Now, we need to assume the existence of a linkage between the pair $\left( {Y_{ \uparrow }^{{({\text{in}})}} \wedge Z_{ \downarrow }^{{{\text{(fin}})}} } \right)$. This linkage is imposed by the physical linkage between the operators $\Im_{{{\text{input}}}} \circ \,\,\Im_{{{\text{output}}}}$. In an enzymatic catalysis, the linkage between variables $Y_{ \uparrow }^{{({\text{in}})}}$ and $Z_{ \downarrow }^{{({\text{fin}})}}$ has chemical and thermodynamic constraints, but in the case of neural associative memories, these a priori constraints do not exist (see Sect. 3.2), because they emerge after contingent associative experiences.

Let us exemplify with some details these Y and Z sets based on the examples in Sect. 3. In the case of enzymatic catalysis (Fig. 1), $Y_{ \downarrow }^{{({\text{in}})}}$ is the set of potential substrates and $Y_{ \uparrow }^{{({\text{in}})}}$ is the subset of substrates selected by the specificity of the active sites of the enzyme; $Z_{ \downarrow }^{{({\text{fin}})}}$ is the set of possible thermodynamically and chemically accessible states from $Y_{ \uparrow }^{{({\text{in}})}}$, and $Z_{ \uparrow }^{{{\text{(fin}})}}$ is the set of transformations selected by the properties of the catalytic sites of the enzyme. (The chemical and thermodynamic basis of this catalytic process is illustrated in detail in Dixon and Webb 1979.) In the case of a neural system, we will describe the interpretation of the sets based on both Fig. 2 and the prisoner's parable: the set $Y_{ \downarrow }^{{({\text{in}})}}$ is the set of total signals received by the system (whether they represent noise or a code) and $Y_{ \uparrow }^{{({\text{in}})}}$ is the subset recognized by the neural module; this $Y_{ \uparrow }^{{({\text{in}})}}$ subset enters a network that is capable of selecting from a potential set of motor acts $Z_{ \downarrow }^{{({\text{fin}})}}$, the specific subset $Z_{ \uparrow }^{{({\text{fin}})}}$ of actions that solve the problem, in the case of the prisoner, the movements that open the safe. (An illustration of how a modular neural system achieves this type of specific response is shown in Mizraji 1989 and Mizraji et al 2009).

Let us assume that a *very complex* system can be characterized by a large set of potential transformations

$$\Omega^{{{\text{POT}}}} = \left\{ {\,\left[ {Y_{ \downarrow }^{{({\text{in}},r)}} \to Z_{ \uparrow }^{{({\text{fin}},q)}} } \right],\,\,\,(r,q) \in {\mathbb{N}} \times {\mathbb{N}}\,} \right\}.$$

(with low transition probabilities in the absence of a iCat). The set $\Omega^{{{\text{POT}}}}$ has a high cardinal number. Let $\Phi = \left\{ {{\text{iCat}}} \right\}$ be the actual set of iCat's that exists inside the system. Let us also assume the existence of a function $\Upsilon$,

$$\Upsilon :\,\,\Omega^{{{\text{POT}}}} \times \,\,\Phi \,\,\, \to \,\,\,\Omega^{{{\text{ACT}}}} ,$$

that represents the collection of transformations from $\Omega^{{{\text{POT}}}}$ onto the very much smaller set $\Omega^{{{\text{ACT}}}} \subset \Omega^{{{\text{POT}}}}$ (the transformations that actually happen), promoted by the set of information catalysts $\Phi$. *This is a formalization of the creation of order by a set of iCat's.*
