# Correction to: The biological Maxwell's demons: exploring ideas about the information processing in biological systems

**DOI:** 10.1007/s12064-021-00356-4

**Published:** 2021-09-21

**Authors:** Eduardo Mizraji

**Affiliations:** Group of Cognitive Systems Modeling, Biophysics and Systems Biology Section, Facultad de Ciencias, Universidad de La República, Iguá 4225, 11400 Montevideo, Uruguay

## Correction to: Theory in Biosciences https://doi.org/10.1007/s12064-021-00354-6

Authors would like to correct the error in their publication.

This last paragraph, previous to Appendix 1, begins with the expression "Maxwell's physical theory of demons …". However, the correct version is "Physical theory of Maxwell's demons …".

The original article has been corrected.

